# Triel Bonds between BH_3_/C_5_H_4_BX and M(MDA)_2_ (X = H, CN, F, CH_3_, NH_2_; M = Ni, Pd, Pt, MDA = Enolated Malondialdehyde) and Group 10 Transition Metal Electron Donors

**DOI:** 10.3390/molecules29071602

**Published:** 2024-04-03

**Authors:** Xin Wang, Zhihao Niu, Sean A. C. McDowell, Qingzhong Li

**Affiliations:** 1 The Laboratory of Theoretical and Computational Chemistry, School of Chemistry and Chemical Engineering, Yantai University, Yantai 264005, China; wangxin19980705@s.ytu.edu.cn (X.W.); nzh19981019@s.ytu.edu.cn (Z.N.); 2 Department of Biological and Chemical Sciences, The University of the West Indies, Cave Hill Campus, Bridgetown BB11000, Barbados

**Keywords:** π–hole, σ–hole, triel bond, transition metal

## Abstract

A systematic theoretical study was conducted on the triel bonds (TrB) within the BH_3_∙∙∙M(MDA)_2_ and C_5_H_4_BX∙∙∙M(MDA)_2_ (M = Ni, Pd, Pt, X = H, CN, F, CH_3_, NH_2_, MDA = enolated malondialdehyde) complexes, with BH_3_ and C_5_H_4_BX acting as the electron acceptors and the square-coordinated M(MDA)_2_ acting as the electron donor. The interaction energies of these systems range between −4.71 and −33.18 kcal/mol. The larger the transition metal center M, the greater the enhancement of the TrB, with σ–hole TrBs found to be stronger than π–hole TrBs. In the σ–hole TrB complex, an electron-withdrawing substituent on the C opposite to the B atom enhances the TrB, while an electron-donating substituent has little effect on the strength of TrB in the Pd and Pt complexes but enhances the TrB in the Ni-containing complexes. The van der Waals interaction plays an important role in stabilizing these binary systems, and its contribution diminishes with increasing M size. The orbital effect within these systems is largely due to charge transfer from the d_z_^2^ orbital of M into the empty p_z_ orbital of B.

## 1. Introduction

The interaction formed between a group 13 element acting as a Lewis acid center and a Lewis base is called a triel bond (TrB) [1], and it may be considered to be a noncovalent interaction. The noncovalent interaction has always been a “hot” research topic, having important applications in molecular assembly [2,3], protein structure regulation [4,5], organic catalysis [6,7,8], molecular recognition [9], and other fields. When triel atoms bond with other atoms, they usually undergo sp^2^ hybridization to form a planar triangular configuration. This special structure and the excellent properties of triel bonds (with a wide range of strength adjustment) give rise to important applications in catalysis [10], crystal material construction [11], dye synthesis [12], hydrogen storage materials [11], and other fields. Due to the electron deficiency of the p_z_ orbitals above the sp^2^-hybridized Tr atom, a positive electrostatic potential develops above the center of the triangular plane, known as a π–hole [13]. This positive region can interact with a series of nucleophilic molecules.

In the 1960s, 1:1 van der Waals dimers were discovered by infrared spectroscopic studies of mixtures of ethylene/propylene and BF_3_ and the electron-deficient site above B bound to the C=C double bond [14]. Subsequent studies found that a similar interaction also existed between BF_3_ and other Lewis bases (NH_3_, HCN, PhCN, N(CH_3_)_3_) [15,16,17]. In addition, spectral and theoretical studies of X-CH_3_CN-BF_3_ (X = F, Cl, Br, I) found that the complex possessed a very weak coordination bond in the gas phase and a very short N-B distance in the solid phase. These all indicated the existence of N-B interactions [18]. In the theoretical study of the TrB between TrF_3_- (Tr = B, Al) and LP-type N-containing electron donors, Grabowski found that some C_3V_ symmetric complexes were formed, where N interacts with B and Al [19,20]. In subsequent research, he defined this interaction as a TrB [1]. This type of interaction is often very strong, and its stability mainly depends on the charge transfer caused by the coordination. The properties of Lewis base centers and the electron-absorbing ability of substituents, as well as the feedback bond effect, are also closely related to the strength of the interaction. Studies have also been conducted on the other group 13 elements. By comparison, the TrB formed by B was found to be the weakest, and different Tr atoms have different properties when used as Lewis acid centers. Michalczyk et al. conducted a systematic study on the factors affecting the strength of TrBs [21]. Theoretical research on the TrB between TrR_3_ (R = H, F, Cl, Br) and pyrazine found that when Tr = Al or Ga, the TrB strength varies with the electron-withdrawing ability of the substituent, with F > Cl > Br > H, whereas for Tr = B, the order is reversed. Orbital interactions play an important role in the formation of TrBs. B-containing systems have the strongest orbital interactions, and the orbital interactions are comparable in magnitude to electrostatic interactions. For the systems containing Al or Ga, the electrostatic interaction plays the dominant role in the formation of TrBs, followed by orbital interactions.

Transition metals (TM) seem anomalous since they often exhibit Lewis acid behavior due to their unfilled d orbitals and the 18-electron rule. The Lewis base properties of transition metals are usually observed in the feedback phenomenon from metal to ligand, but TMs can exhibit pure Lewis base behavior when interacting with Lewis acids [22,23]. In the 1960s, it was first proposed that metals could act as hydrogen-bond acceptors based on the liquid-phase infrared spectroscopy of ferrocene alcohols [24,25,26,27]. At the same time, the existence of interactions between boranes and metals was also predicted, but the structures were not validated [28,29]. In 1979, Burlitch and his colleagues reported the first evidence of the interaction between TM and triel atoms, obtained via crystallographic confirmation. They found that Al in AlPh_3_ can coordinate with Fe in [FeCp(CO_2_)]^−^ [30]. Subsequently, a crystallographic study confirmed the interaction between heavier Tr atoms (Al, Ga, In) and TM [31], accompanied by short TM-Tr distances and a clear pyramiding of the chemical environment around the Tr atoms. The TrBs formed by B and TM are weak, making it difficult to form stable dimers between B and TM. With the development of Lewis acid-base strategies with rigid multi-toothed frameworks in 1999, Hill and his colleagues synthesized and validated the first pillar TM-B complex. The shorter Ru-B distance and the chemical environment surrounding B showed the presence of a Ru-B interaction [32].

The group 10 transition metals are relatively common TrB electron donors. A study on the interaction between [Pt(PCy_3_)_2_] and the heavier Tr atoms shows that Al atoms can directly coordinate with Pt [33]. For Ga, the binding form of the complex depends on the properties of the halogen ligand. Among the three Lewis acids, GaCl_3_, GaBr_3_, and GaI_3_, only GaCl_3_ forms the target complex with [Pt(PCy_3_)_2_] [34]. X-ray diffraction confirmed the T-shaped geometric shape formed by the P-Pt-P framework and Tr atoms in the corresponding complexes of AlCl_3_ and GaCl_3_. Theoretical work on [TM(PMe_3_)_2_(TrX_3_)] (TM = Ni, Pd, Pt, Tr = F, Cl, Br, I, X = F, Cl, Br, I) provided more information on this interaction. The interaction between B and TM is the weakest, with the dissociation energy not exceeding 27 kcal/mol. The metallaboratranes with a triangular biconical structure for Ni [35], Pd, Pt [36] were prepared via a rigid multi-toothed framework strategy. The three common ligands of TM and B fix TM directly above the B, and the chemical environment around B undergoes significant tapering. The influence of metal centers on the TM–B interaction has also been studied. In the d^8^ DPB composite, the position of the ^11^B NMR signal in the Pt system is higher than that of Pd, indicating that the interaction with Pd is weaker than with Pt. The TM-B binding distance also indicates the same issue, with Pd having a longer TM-B binding distance and a significant decrease in NBO delocalization energy [23].

For the crystal research on d^8^ Ni, it was found that Ni can act as an electron donor to form Ni∙∙∙I halogen bonds, with a halogen bond strength of 4.5 kcal/mol [37,38]. Further research showed that Pd and Pd could also be considered as halogen bond receptors [39]. Subsequently, various types of TM∙∙∙I interactions were identified [40,41,42,43]. Ni and Pt metal nanoparticles can interact with Na^+^, HF, and H_2_O, due to the attraction between local positive and negative electrostatic potential regions on the two monomers. On this basis, the author also demonstrated the correlation between electrostatic potential and binding energy [44]. Recently, Zierkiewicz et al. investigated the ability of Pt and Pd atoms in square–planar coordination geometries to act as σ–hole acceptors. The σ–hole bond is quite strong and varies in the range 6 to 20 kcal/mol. The σ–hole bond involving Pt is stronger than the corresponding bond for Pd, and this difference increases as the size of the Lewis acid atom increases [45]. The π–hole interaction between C_6_F_6_ and the square–planar [M(II)] (M = Pd, Pt) coordination complex has also been studied. There is a weak negative potential region on the M surface, and the bond critical point between C∙∙∙M in QTAIM analysis indicates the presence of a [M(II)]∙∙∙π–hole interaction [46]. Malenov et al. investigated the stacking interactions between acac-type chelates of Ni, Pd, and Pt, where the ligands in these chelates are formate anions and enolated forms of malondialdehyde. The stacking interaction between chelates ranges from −9.21 to −9.70 kcal/mol, and the strength of the interaction increases with an increase in the metal’s atomic number [47]. They also investigated the stacking interactions of acac-type chelates of Ni, Pd, Pt with benzene. The stacking interaction ranges from −5.36 to −5.75 kcal/mol, and the strength of the interaction decreases with an increase in the metal’s atomic number [48].

To the best of our knowledge, there are currently no reports on the use of group 10 metals in square–planar coordination geometries acting as triel bond acceptors. In addition, although the π–hole interaction between the group 13 elements and M (M = Ni, Pd, Pt) with linear geometric coordination has been studied before [49], the presence of strong secondary interactions between halogen ligands and M causes a significant twist in the target structure of the T-type, thus making it difficult for the full strength of the Tr∙∙∙M interaction to be manifested. In order to reduce the influence of secondary interactions and maintain good directionality between B∙∙∙M, we used [M(II)] atoms coordinated in a square–planar geometry as electron donors interacting with BH_3_ in order to more accurately study π–hole Tr∙∙∙M interactions. In the present work, the triel bonds in the dimers of BH_3_∙∙∙M (MDA)_2_ and C_5_H_4_BX∙∙∙M (MDA)_2_ (M = Ni, Pd, Pt, X = H, CN, F, CH_3_, NH_2_, MDA = enolated malondialdehyde) were systematically studied. To investigate substituent effects on the strength of triel bonds, the H on the para-C was replaced by different functional groups in C_5_H_5_B. The properties of triel bonds in the system were studied, and their formation mechanism was explained through structural analysis, interaction energy calculations, AIM, IRI, and NOCV analyses.

## 2. Results

### 2.1. MEP Analysis

**Figure 1:**
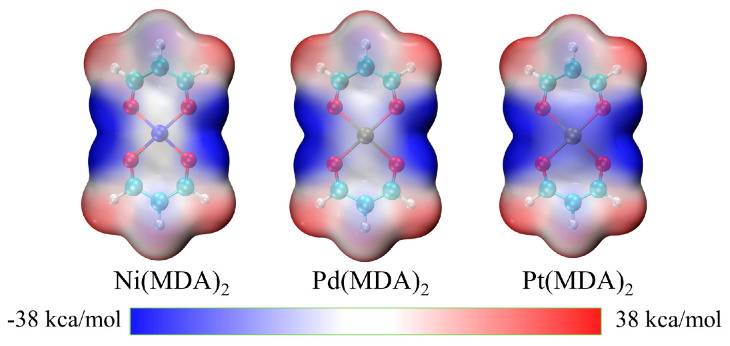
The MEP maps on the 0.001 electrons Bohr^−3^ surface of M(MDA)_2_ (M = Ni, Pd Pt). Red and blue regions represent positive and negative MEPs, respectively.

Figure 1 shows the electrostatic potential maps for M(MDA)_2_ (M = Ni, Pd Pt), while those of BH_3_ and C_5_H_4_BX (X = H, CH_3_) are plotted in Figure S1. Table 1 collects the most positive electrostatic potential value (V_s,max_) corresponding to the π–hole on BH_3_ and σ–hole on C_5_H_4_BX. From the results, the π–hole is weaker than the σ–hole. For C_5_H_4_BX, the presence of electron-withdrawing groups increases the V_s,max_ values, and this increase in value is positively correlated with its electron-withdrawing ability. The introduction of electron-donating groups has little effect on V_s,max_ values. Although the most negative electrostatic potential value was not found in the metal center, it can be seen from the figure that there is a weak negative potential region above the metal atom, and this region eventually turns blue as the atomic mass of M increases. This means that the heavier M Lewis base center has a more negative electrostatic potential region, which may form stronger triel bonds than does the lighter M center.

### 2.2. Structural Analyses

**Figure 2:**
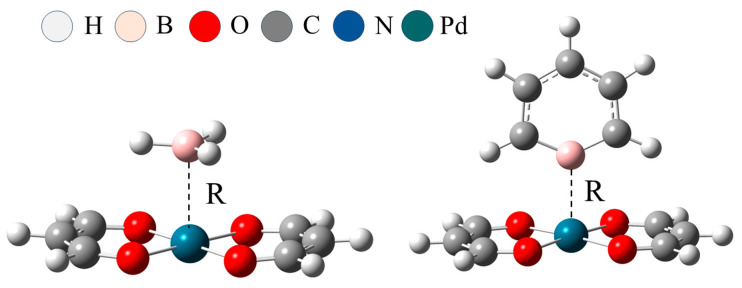
Optimized structures of BH_3_∙∙∙Pd(MDA)_2_ (**left**) and C_5_H_5_B∙∙∙Pd(MDA)_2_ (**right**), marked with the B∙∙∙M distance (R, Å).

All B atoms in the system are stably bound above the metal atoms, and there is a clear directionality between B∙∙∙M. Figure 2 is a schematic diagram of the structure of BH_3_∙∙∙Pd(MDA)_2_ and C_5_H_5_B∙∙∙Pd(MDA)_2_. For the π–hole system, all system configurations are similar to BH_3_∙∙∙Pd(MDA)_2_, with the BH_3_ plane parallel to the M(MDA)_2_ plane. The configuration of the σ–hole system is similar to that of C_5_H_5_B∙∙∙Pd(MDA)_2_, with the line connecting the para-C and B pointing towards the metal atom and perpendicular to the M(MDA)_2_ plane. Table 2 shows that the B∙∙∙M binding distance R in the π–hole system is longer than in the σ–hole system. All B∙∙∙M binding distances lie between the sum of the covalent radii of B and M and the sum of their van der Waals radii, and vary with a change of X substituents. The introduction of electron-withdrawing substituents shortens the B∙∙∙M binding distance, and the greater the electron withdrawing-ability of X, the greater the shortening of the B∙∙∙M binding distance. The addition of electron-donating groups also shortens the binding distance between B and M but to a lesser extent. For the Ni-centered system, substitution at the para-C position in C_5_H_5_B by the -CH_3_ and -NH_2_ groups shortens the B∙∙∙M binding distance by about 0.18 Å; the B∙∙∙M distances in C_5_H_4_BCH_3_∙∙∙Ni(MDA)_2_ and in C_5_H_4_BNH_2_∙∙∙Ni(MDA)_2_ are 2.604 Å and 2.611 Å, respectively, differing by no more than 0.007 Å. However, substitution of the -CH_3_ and -NH_2_ in the Pd and Pt systems has little effect on the B∙∙∙M binding distance, which does not change by more than 0.01 Å. The B∙∙∙M binding distance is generally in the order C_5_H_4_BCN < C_5_H_4_BF < C_5_H_5_B < C_5_H_4_BNH_2_ < C_5_H_4_BCH_3_ < BH_3_. For fixed Lewis acid, the heavier metal centers correspond to a shorter binding distance, indicating enhancement of the corresponding TrB.

### 2.3. Interaction Energy

In addition to the B∙∙∙M binding, the interaction energy E_int_, binding energy E_b_, and deformation energy DE between all monomers in the complexes were also calculated. Both E_int_ and E_b_ display similar variations. The interaction energy ranges between −4.71 and −33.18 kcal/mol. For the fixed Lewis acid, E_int_ increases as M gets larger, which is consistent with the results for the B∙∙∙M binding distance and electrostatic potential analysis; i.e., the heavier group 10 metals tend to form stronger TrBs. For the fixed M, the interaction energy increases and the B∙∙∙M binding distance decreases with an increase in the electron-withdrawing ability of the X substituent; i.e., when M = Ni, C_5_H_4_BCN > C_5_H_4_BF > C_5_H_4_BNH_2_ > C_5_H_4_BCH_3_ > C_5_H_5_B > BH_3_ for E_int_ and the reverse order for the B∙∙∙M distance, whereas when M = Pd or Pt, C_5_H_4_BCN > C_5_H_4_BF > C_5_H_4_BNH_2_ > C_5_H_5_B > C_5_H_4_BCH_3_ > BH_3_ for E_int_ and the reverse order for the intermolecular separation, with the computed values for C_5_H_5_B ≈ C_5_H_4_BCH_3_ ≈ C_5_H_4_BNH_2_. The σ–hole TrB is thus stronger than the π–hole TrB, and the electron-withdrawing substituents enhance the system’s interaction energy, while the electron-donating substituents have little effect. The deformation energy DE ranges from 0.24 to 7.96 kcal/mol, and also increases with the atomic mass of M, indicating that systems with heavier metal centers undergo greater polarization and have stronger orbital interactions.

### 2.4. AIM Analysis

**Figure 3:**
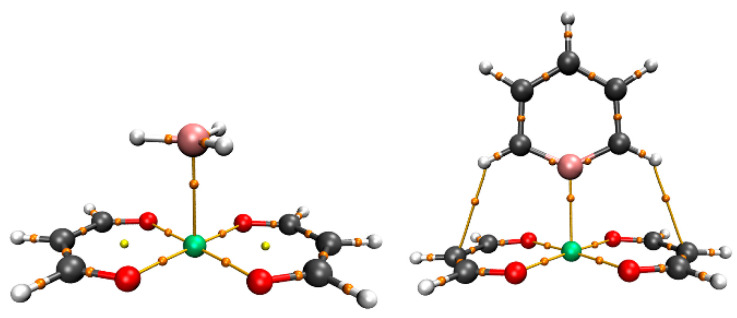
AIM diagrams of BH_3_∙∙∙Pd(MDA)_2_ (**left**) and C_5_H_5_B∙∙∙Pd(MDA)_2_ (**right**); bond paths are indicated by broken lines with bond critical point represented by small orange dots.

In order to further verify the presence of TrBs in the complexes and explore their properties, we conducted AIM analyses on all dimers. Figure 3 shows the AIM diagram of two systems. Taking BH_3_∙∙∙Pd(MDA)_2_ and C_5_H_5_B∙∙∙Pd(MDA)_2_ as examples, there are clear bond critical points (BCPs) between all B∙∙∙M. With reference to the σ–hole system, in addition to the bond path of the target TrB, there are also two other bond paths between H∙∙∙C, connecting the two H atoms on the C atoms adjacent to and on either side of B to the two C atoms on the (MDA)_2_ ring to which they point, indicating the existence of secondary interactions such as H-bonds in these systems. Table 3 shows the electron density of the B∙∙∙M BCP (ρ), the Laplacian of the electron density (∇^2^ρ), and the total energy density (H). The electron density ρ increases as M gets larger, and ρ for the π–hole system is smaller than for the σ–hole system. The electron-withdrawing substituents increase ρ at the BCP, but there is little to no change in ρ due to the electron-donating groups (CH_3_, NH_2_). At the BCP in the Ni systems, ∇^2^ρ is positive and H negative, indicating that their TrBs exhibit partially covalent characteristics. For the Pd and Pt systems, except for BH_3_∙∙∙Pd(MDA)_2_ and BH_3_∙∙∙Pt(MDA)_2_ (where ∇^2^ρ is positive and H is negative), both ∇^2^ρ and H are negative, indicating that the TrBs in these systems have covalent characteristics.

### 2.5. IRI Analysis

**Figure 4:**
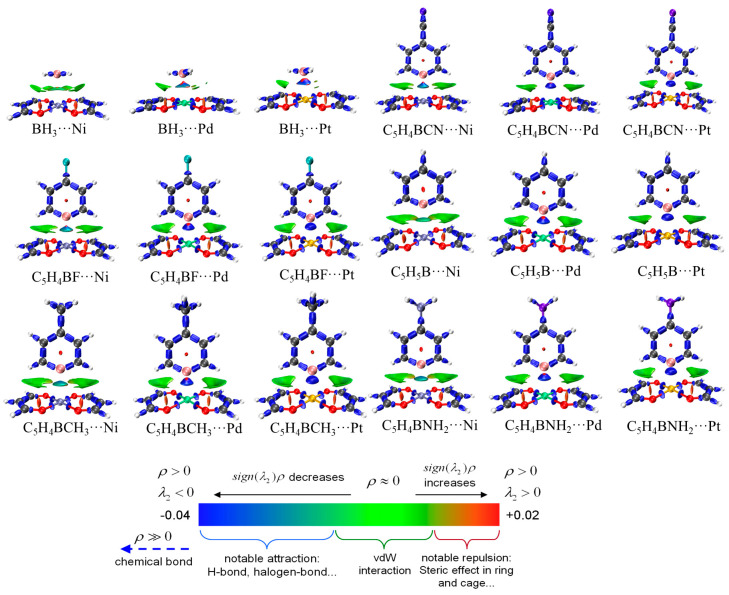
IRI diagrams of BH_3_∙∙∙M(MDA)_2_ and C_5_H_4_BX∙∙∙M(MDA)_2_ (M = Ni, Pd, Pt, X = H, CN, F, CH_3_, NH_2_).

In order to study the interactions within the system more intuitively, we also conducted IRI analyses to visualize the interactions within the system. Figure 4 shows the IRI diagram of the systems. The isosurface between B∙∙∙M continuously turns blue with an increase in the M atomic mass, and for the systems containing BH_3_, this change is most pronounced, going from dark green between B∙∙∙Ni to blue between B∙∙∙Pd and then to dark blue between B∙∙∙Pt. This suggests that the TrB of these three systems transitions from interactions slightly stronger than those due to van der Waals forces to moderately strong interactions and then finally to strong interactions closer in strength to covalent bonds. In C_5_H_4_BX∙∙∙M(MDA)_2_, two light green isosurfaces on both sides of the target TrB are evident, which correspond to the bond path between H∙∙∙C in AIM analysis, thereby confirming the existence of secondary H-bond interactions. However, from the perspective of color, their intensities are small and comparable to van der Waals forces.

### 2.6. EDA Analysis

In order to assess the energetic contributions to B∙∙∙M TrBs, we conducted EDA analysis on all systems, which decomposed the interaction energy into six terms: electrostatic energy E^ele^, exchange energy E^ex^, repulsion energy E^rep^, polarization energy E^pol^, dispersion energy E^disp^, and electron correlation energy E^corr^. Table 4 presents these data. The total interaction energy obtained from the EDA method has almost the same magnitude as that obtained from the supermolecular method, suggesting that each energy term obtained by EDA is reliable. The E^rep^ term has a relatively large positive value and correlates with decreasing Tr∙∙∙M distance as M gets larger. On the other hand, the exchange energy is the largest attractive term, usually coexisting with the E^rep^ term (confirmed by the strong linear relationship shown in Figure S2), which causes both terms to offset each other, thereby diminishing their net effect on the interaction energy; thus, these terms are not discussed in detail. No good linear relationship is obtained between the total interaction energy and each term; thus, there appears to be no consistent trend for each energetic term in all complexes. The sum of E^disp^ and E^corr^ reflects the van der Waals interaction, while the orbital interaction is partly reflected by E^pol^. The van der Waals interaction dominates in the Ni and Pd systems, followed by the electrostatic interaction, with the orbital interaction being the weakest. The variation of each energetic term in the Pt systems is not straightforward. Most Pt systems are dominated by the orbital interaction, and the van der Waals interactions are weakest, while the reverse result is found for BH_3_∙∙∙Pt(MDA)_2_. As M gets larger, the proportion of electrostatic and orbital interactions contributing to the TrB strength increases, whereas the E^disp/corr^ variation (for the same Lewis acid) is irregular.

### 2.7. NOCV Analysis

**Figure 5:**
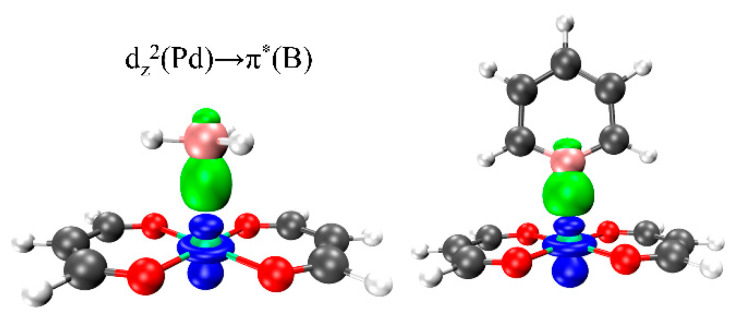
NOCV diagram of BH_3_∙∙∙Pd(MDA)_2_ (**left**) and C_5_H_4_BCN∙∙∙Pd(MDA)_2_ (**right**); the green electron cloud represents accepted electrons, while the blue electron cloud represents donated electrons. The symbol * denotes the empty B orbital.

Figure 5 shows the density difference plot of NOCV in the complexes, and the orbital effect of TrB in all systems, which mainly comes from the charge transfer from the d_z_^2^ orbital of M into the empty p_z_ orbital of B. Table 5 shows the NOCV energy in different systems. From the table, it can be seen that the NOCV energy of the σ–hole system is stronger than that of the π–hole system. The electron-withdrawing substituents increase the NOCV energy, and do so to a much greater extent than do the electron-donating substituents, relative to the C_5_H_5_B. In fact, for the Pd and Pt systems, there is hardly any change in the NOCV energy due to CH_3_ or NH_2_ substitution in C_5_H_5_B. For all complexes, as M goes from Ni to Pd to Pt, the NOCV energy increases continuously. Thus, overall, electron-withdrawing substituents and heavier metal centers are more conducive to charge transfer between monomers, while electron-donating substituents only promote charge transfer in the Ni systems and have little effect on the other systems.

## 3. Discussion

The MEP analysis shows that there is a weak negative potential region in the center of the group 10 metal square–planar moiety, which increases with an increase in M atomic mass. Rozhkov et al. studied the electrostatic potential of similar structures [46], but their research focused only on Pd and Pt metal centers; their conclusions are consistent with those from this present work. Although the most negative electrostatic potential value was not obtained, the weak negative potential region at the metal center still gives a good indication of the possible B∙∙∙M binding site.

In the study of B∙∙∙M interactions, the possibility of large secondary effects can often be a complicating factor since they may cause displacement of the preferred B∙∙∙M target binding site [49]. In this work, there is a clear directionality between B∙∙∙M in all designed dimers, with the B atom located above the M atom. The B∙∙∙M binding distance R ranges between 2.233 and 2.842 Å, these values all lying between the sum of the van der Waals radii of B and M and the sum of their covalent radii, which conforms to a defining characteristic of noncovalent interactions. For the fixed Lewis acid, R decreases with an increase in the M atomic mass, indicating that heavier metal centers are more conducive for the enhancement of TrBs and consistent with the MEP results. For the fixed M, the binding distance between B and M exhibits two effects as the type of X substituent changes. Firstly, electron-withdrawing substituents cause a decrease in the B∙∙∙M binding distance with the increase in electron-withdrawing ability, consistent with the MEP analysis. These types of substituents reduce the electron density on the six-membered Lewis acid ring, thereby increasing the magnitude of the σ–hole; consequently, the greater the electron-withdrawing ability of the substituent, the stronger the TrB.

Surprisingly, the σ–hole should have been diminished by the presence of the electron-donating substituents, but their influence was found to be negligible in the Pd and Pt systems, where the B∙∙∙M binding distance hardly changes (decreasing by no more than 0.003 Å). However, in the Ni systems, the binding distances are significantly shortened; for example, the B∙∙∙Ni distance in C_5_H_4_BCH_3_∙∙∙Ni(MDA)_2_ is shortened by 0.182 Å relative to C_5_H_4_B∙∙∙Ni(MDA)_2_. The B∙∙∙M binding distance in BH_3_∙∙∙M(MDA)_2_ is longer than in C_5_H_4_BX∙∙∙M (MDA)_2_, which means that the π–hole B∙∙∙M TrB is weaker than the σ–hole B∙∙∙M TrB.

The interaction energy of the B∙∙∙M TrB ranges between −4.71 and −33.18 kcal/mol, spanning weak and strong noncovalent interactions. The interaction energy increases as the central metal atom gets larger, consistent with the MEP and structural analyses, as mentioned earlier. The π–hole interaction energy is smaller in magnitude than the σ–hole interaction energy. For the fixed Lewis base, electron-withdrawing substituents enhance the interaction energy, while electron-donating substituents hardly change the interaction energy.

**Figure 6:**
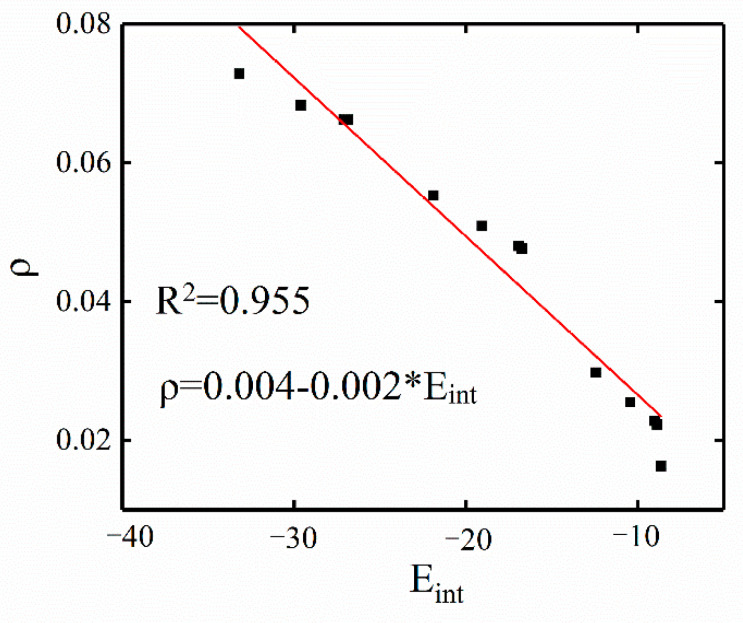
Linear relationship between the electron density (ρ, a.u.) at the B∙∙∙M BCP and the interaction energy (E_int_, kcal/mol) in C_5_H_4_BX∙∙∙M(MDA)_2_.

AIM analysis is one of the powerful tools for studying noncovalent interactions. AIM analysis shows that, in addition to the presence of BCPs between the target B∙∙∙M region, there are also two other BCPs in the C_5_H_4_BX systems, which correspond to two weak hydrogen-bonding secondary interactions. The interaction energy is the sum of all interaction strengths within the system. Will the presence of secondary interactions hinder the accurate description of the target TrB’s binding strength? In previous studies, good correlations between the electron density at the BCP and the corresponding interaction strength were reported [50,51], so the correlation between the TrB and the interaction energy can be used to measure the impact of secondary interactions on the total interaction energy. Figure 6 shows a good linear relationship between the electron density (ρ) at the B∙∙∙M BCP in C_5_H_4_BX∙∙∙M(MDA)_2_ and interaction energy (E_int_). The scatter plot shows a very good correlation between E_int_ and ρ, with a correlation coefficient of 0.96, indicating that the contribution of secondary effects to the total interaction energy is very small.

The results of the IRI analysis and the AIM analysis are consistent with each other, and the presence of two light green regions at the weak hydrogen bond positions in the C_5_H_4_BX system also indicates that the secondary effect is quite weak. There is a clear density gradient isosurface between B∙∙∙M, which further indicates the presence of a TrB. The isosurface turns blue with the increase in M atomic mass, indicating enhancement of the TrB. The large green isosurfaces in the Ni system indicate the important role of the van der Waals interaction in the formation of its TrB.

The type of interaction can also be judged from the results of the EDA analysis. For example, for tetrel bonds formed by C_60_ and SnI_4_ [52] and triangular coinage metal complexes of (M_3_ = Cu, Ag, and Au) and C_60_ [53], the dispersion energy makes the largest contribution, followed by electrostatic energy, suggesting a van der Waals nature; however, for N-heterocyclic carbene(NHC)-M∙∙∙OEt_2_ complexes (M = Cu, Ag, Au) [54] and NHC-M pyrazine bond (M = Cu, Ag, Au) interactions [55], the contribution of electrostatic energy exceeds 60%, and orbital interaction is close to 30%; thus, these interactions show electrostatic character. In this paper, EDA results show that a van der Waals interaction is dominant in the formation of TrBs in the Ni/Pd-containing systems (electrostatic and orbital interactions are more important in the Pd systems than in the Ni systems). In the Pt systems, orbital interactions dominate, followed by electrostatic interactions, with the van der Waals interactions being the weakest.

The unexpected positive values obtained by EDA for the polarization energy contribution to the interaction energy in the Ni systems may be due to the introduction of the electron correlation energy term. Compared to LMO-EDA [56], a portion of the newly added E_corr_ term in the GKS-EDA method comes from the E_pol_ term. The orbital interaction in the Ni system is weak, and after deducting the E_corr_ term, E_pol_ becomes positive. As the atomic mass of M increases, the proportion of electrostatic and orbital interactions contributing to the interaction energy increases, ultimately surpassing the contribution of the van der Waals interactions. The NOCV analysis confirms this phenomenon, showing that the NOCV energy increases as M gets larger, which indicates that the orbital interaction between the systems is being enhanced accordingly (Table 5). The orbital interaction between the systems mainly comes from the charge transfer from the d_z_^2^ orbital on M into the p_z_ orbital of B.

## 4. Theoretical Methods

All calculations were performed using the Gaussian suite of programs [57] at the B3LYP-D3(BJ)/aug-cc-pVTZ(PP) [58,59,60] level of theory, using the basis set aug-cc-pVTZ-PP for Pd, Pt, and aug-cc-pVTZ for the other atoms. All structures were fully optimized, with the absence of any imaginary frequencies confirmed by vibrational frequency calculations at the same level. The interaction energy and binding energy were calculated using the supermolecular method, and the basis set superposition error (BSSE) correction was performed using the equilibrium method proposed by Boys and Bernardi [61]. The Multiwfn program [62] was used to perform topological analysis of the bond critical point (BCP) electron density of the complexes using the atoms in molecules (AIM) theory [63]. The interaction region indicator function (IRI) method [64] was used to visualize the TrBs in the complexes. The orbital interactions in the complexes were analyzed using the Extended Transition State-Natural Orbitals for Chemical Valence (ETS-NOCV) method [65]. IRI and ETS-NOCV analyses also utilized the Multiwfn program [62]. In order to predict the binding site, the molecular electrostatic potential (MEP) of the monomers on the 0.001 a.u. electron density isosurface was analyzed using the Multiwfn program [62]. Energy decomposition calculations were performed using XEDA [66] software (https://xacs.xmu.edu.cn, accessed on 10 January 2024) combined with GKS-EDA [67] theory, and XEDA input files were generated using the Mokit program [68].

## 5. Conclusions

A systematic theoretical study was conducted on the triel bonds within BH_3_∙∙∙M(MDA)_2_ and C_5_H_4_BX∙∙∙M(MDA)_2_ complexes at the B3LYP-D3 (BJ)/aug-cc-pVTZ(PP) level. The interaction energy of the system ranges between −4.71 and −33.18 kcal/mol and can be regulated so as to span the two extremes of weak and strong noncovalent interactions. The TrB is enhanced by an increase in the size of the metal center M, with the σ–hole TrB found to be stronger than the π–hole TrB. For the σ–hole TrB complexes, electron-withdrawing substituents at the C position para to B strengthen the B∙∙∙M TrB, while electron-donating substituents have little effect in the Pd and Pt complexes, but have a significant enhancing effect in the Ni complexes. Weak hydrogen bonds are present as secondary interactions in the σ–hole TrB systems; however, their contribution to the net binding is insignificant. The van der Waals force plays an important role in stabilizing these systems, and dominates in the Ni complexes, with its contribution to the interaction energy consistently diminishing as the metal gets larger. In the Pd system, it is comparable in magnitude to the orbital and electrostatic contributions, while in the Pt system, the orbital interactions dominate. The orbital interaction arises mainly from the charge transfer from the d_Z_^2^ orbital of M into the empty p_z_ orbital of B, which increases as the metal gets larger, going from Ni to Pd to Pt.

## Figures and Tables

**Table 1:** The most positive MEP (V_s,max_, kcal/mol) on the π–hole of BH_3_ and the σ–hole of C_5_H_4_BX (X = H, CN, F, CH_3_, NH_2_).

	V_s,max_
BH_3_	41.35
C_5_H_5_B	44.07
C_5_H_4_BF	52.08
C_5_H_4_BCN	59.58
C_5_H_4_BCH_3_	43.35
C_5_H_4_BNH_2_	44.02

**Table 2:** Binding distance (R, Å), interaction energy (E_int_, kcal/mol), binding energy (E_b_, kcal/mol), and deformation energy (DE, kcal/mol) in the complexes.

	R	E_int_	E_b_	DE
BH_3_-Ni	2.842	−4.71	−4.47	0.24
BH_3_-Pd	2.544	−8.60	−7.56	1.04
BH_3_-Pt	2.447	−14.09	−11.38	2.71
C_5_H_5_B-Ni	2.786	−8.66	−8.00	0.66
C_5_H_5_B-Pd	2.356	−16.76	−13.16	3.60
C_5_H_5_B-Pt	2.271	−26.91	−20.17	6.74
C_5_H_4_BF-Ni	2.528	−10.44	−8.85	1.59
C_5_H_4_BF-Pd	2.323	−19.07	−15.02	4.05
C_5_H_4_BF-Pt	2.254	−29.61	−22.61	7.00
C_5_H_4_BCN-Ni	2.466	−12.46	−10.30	2.15
C_5_H_4_BCN-Pd	2.293	−21.89	−17.08	4.80
C_5_H_4_BCN-Pt	2.233	−33.18	−25.22	7.96
C_5_H_4_BCH_3_-Ni	2.604	−8.88	−7.71	1.17
C_5_H_4_BCH_3_-Pd	2.354	−16.72	−13.16	3.56
C_5_H_4_BCH_3_-Pt	2.269	−26.89	−20.20	6.69
C_5_H_4_BNH_2_-Ni	2.611	−9.03	−7.86	1.18
C_5_H_4_BNH_2_-Pd	2.346	−16.93	−13.38	3.55
C_5_H_4_BNH_2_-Pt	2.265	−27.11	−20.54	6.57

**Table 3:** Electron densities (ρ), Laplacians (∇^2^ρ), and total energy densities (H) at the B∙∙∙M BCP in the complexes, all in a.u.

	ρ	∇^2^ρ	H
BH_3_-Ni	0.0130	0.0264	−0.0004
BH_3_-Pd	0.0301	0.0354	−0.0064
BH_3_-Pt	0.0437	0.0200	−0.0162
C_5_H_5_B-Ni	0.0163	0.0196	−0.0018
C_5_H_5_B-Pd	0.0476	−0.0067	−0.0205
C_5_H_5_B-Pt	0.0662	−0.0489	−0.0427
C_5_H_4_BF-Ni	0.0255	0.0140	−0.0059
C_5_H_4_BF-Pd	0.0509	−0.0156	−0.0244
C_5_H_4_BF-Pt	0.0683	−0.0502	−0.0463
C_5_H_4_BCN-Ni	0.0298	0.0100	−0.0079
C_5_H_4_BCN-Pd	0.0553	−0.0268	−0.0282
C_5_H_4_BCN-Pt	0.0728	−0.0640	−0.0509
C_5_H_4_BCH_3_-Ni	0.0223	0.0179	−0.0041
C_5_H_4_BCH_3_-Pd	0.0477	−0.0070	−0.0208
C_5_H_4_BCH_3_-Pt	0.0662	−0.0476	−0.0431
C_5_H_4_BNH_2_-Ni	0.0228	0.0193	−0.0040
C_5_H_4_BNH_2_-Pd	0.0480	−0.0082	−0.0217
C_5_H_4_BNH_2_-Pt	0.0662	−0.0440	−0.0439

**Table 4:** Electrostatic (E^ele^), exchange (E^ex^), repulsion (E^rep^), polarization (E^pol^), sum of dispersion and electron correlation (E^disp/corr^), and total interaction energies (E^total^) in the complexes, all in kcal/mol.

	E^ele^	E^ex^	E^rep^	E^pol^	E^disp^	E^corr^	E^disp/corr^	E^total^
BH_3_-Ni	−3.13	−14.5	22.46	4.64	−4.56	−9.56	−14.12	−4.65
BH_3_-Pd	−9.47	−35.26	58.77	−2.38	−5.64	−14.52	−20.16	−8.50
BH_3_-Pt	−18.24	−54.52	94.45	−15.85	−5.95	−13.87	−19.82	−13.98
C_5_H_5_B-Ni	−6.03	−21.62	35.08	3.38	−8.26	−11.12	−19.38	−8.58
C_5_H_5_B-Pd	−16.20	−55.47	96.10	−13.02	−9.74	−18.28	−28.02	−16.61
C_5_H_5_B-Pt	−28.82	−80.19	143.69	−37.28	−10.31	−13.83	−24.14	−26.73
C_5_H_4_BF-Ni	−6.53	−30.29	50.21	5.07	−8.28	−20.51	−28.79	−10.34
C_5_H_4_BF-Pd	−16.61	−57.22	99.85	−15.62	−9.85	−19.47	−29.32	−18.92
C_5_H_4_BF-Pt	−28.93	−80.15	144.23	−39.92	−10.36	−14.30	−24.66	−29.43
C_5_H_4_BCN-Ni	−7.79	−33.63	56.56	4.27	−8.44	−23.31	−31.75	−12.34
C_5_H_4_BCN-Pd	−17.91	−59.75	105.48	−19.38	−10.02	−20.16	−30.18	−21.73
C_5_H_4_BCN-Pt	−30.47	−82.44	149.99	−45.72	−10.48	−13.88	−24.36	−33.00
C_5_H_4_BCH_3_-Ni	−6.71	−28.17	46.32	4.70	−8.16	−16.76	−24.92	−8.78
C_5_H_4_BCH_3_-Pd	−16.19	−55.68	96.38	−12.93	−9.76	−18.40	−28.16	−16.57
C_5_H_4_BCH_3_-Pt	−28.80	−80.38	143.89	−37.06	−10.34	−14.02	−24.36	−26.71
C_5_H_4_BNH_2_-Ni	−8.22	−29.64	49.03	3.90	−8.17	−15.84	−29.32	−8.93
C_5_H_4_BNH_2_-Pd	−16.12	−56.16	97.17	−13.27	−9.75	−18.65	−28.40	−16.78
C_5_H_4_BNH_2_-Pt	−28.68	−80.53	143.94	−37.01	−10.33	−14.32	−24.65	−26.93

**Table 5:** NOCV orbital energies (E, kcal/mol) in the complexes.

	Ni	Pd	Pt
BH_3_	−4.75	−14.07	−23.04
C_5_H_5_B	−5.73	−22.70	−34.99
C_5_H_4_BF	−12.29	−25.32	−37.06
C_5_H_4_BCN	−15.03	−28.27	−40.35
C_5_H_4_BCH_3_	−9.47	−22.74	−35.01
C_5_H_4_BNH_2_	−9.05	−23.16	−35.21

## Data Availability

The data presented in this study are available in the article and the Supplementary Materials.

## Supplementary Materials

The following supporting information can be downloaded at: https://www.mdpi.com/article/10.3390/molecules29071602/s1, Figure S1. The MEP maps on the 0.001 electrons Bohr^−3^ surface of the Lewis acids. Red and blue regions represent positive and negative MEPs, respectively. Figure S2. Linear relationship between the exchange energy (E^ex^, kcal/mol) and the repulsion energy (E^rep^, kcal/mol) in BH_3_/C_5_H_4_BX∙∙∙M(MDA)_2_.

## References

[1] Grabowski S.J. (2015). π-Hole bonds: Boron and aluminum Lewis acid centers. ChemPhysChem.

[2] Liu K., Kang Y.T., Wang Z., Zhang X. (2013). Reversible and adaptive functional supramolecular materials: “Noncovalent interaction” matters. Adv. Mater..

[3] Karabıyık H., Ocak, İskeleli N. (2012). Hydrogen-bridged chelate ring-assisted π-stacking interactions. Acta Crystallogr. Sect. B Struct. Sci..

[4] Mundlapati V.R., Sahoo D.K., Bhaumik S., Jena S., Chandrkar A.S., Biswal H. (2018). Noncovalent carbon-bonding interactions in proteins. Angew. Chem. Int. Ed..

[5] Walker M.G., Mendez C.G., Ho P.S. (2023). Non-classical non-covalent σ-hole interactions in protein structure and function: Concepts for potential protein engineering applications. Chem.–Asian J..

[6] de las Nieves-Piña M., Frontera A., Mooibroek T.J., Bauza A. (2021). Frustrated Lewis pairs based on carbon∙∙∙carbon^+^ tetrel bonds: A DFT study. ChemPhysChem.

[7] Wang W., Li X.X., Zhou P.P., Wang Y. (2021). Catalysis with supramolecular carbon-bonding interactions. Angew. Chem. Int. Ed..

[8] Wang J.Z., Young T.A., Duarte F., Lusby P.J. (2020). Synergistic noncovalent catalysis facilitates base-free Michael addition. J. Am. Chem. Soc..

[9] Park S., Kim H.J. (2010). Highly activated Michael acceptor by an intramolecular hydrogen bond as a fluorescence turn-on probe for cyanide. Chem. Commun..

[10] Bhunya S., Malakar T., Ganguly G., Paul A. (2016). Combining protons and hydrides by homogeneous catalysis for controlling the release of hydrogen from ammonia–borane: Present status and challenges. ACS Catal..

[11] Hamilton C.W., Baker R.T., Staubitz A., Manners I. (2009). B–N compounds for chemical hydrogen storage. Chem. Soc. Rev..

[12] Bismillah A.N., Aprahamian I. (2021). Fundamental studies to emerging applications of pyrrole-BF_2_(BOPHY) fluorophores. Chem. Soc. Rev..

[13] Bauzá A., Mooibroek T.J., Frontera A. (2015). The bright future of unconventional σ/π-hole interactions. ChemPhysChem.

[14] Herrebout W.A., Van der Veken B.J. (1997). Van der Waals complexes between unsaturated hydrocarbons and boron trifluoride: An infrared and ab Initio study of etheneBF_3_ and propeneBF_3_. J. Am. Chem. Soc..

[15] Fiacco D.L., Mo Y., Hunt S.W., Ott M.E., Roberts A., Leopold K.R. (2001). Dipole moments of partially bound Lewis acid−base adducts. J. Phys. Chem. A.

[16] Reeve S.W., Burns W.A., Lovas F.J., Suenram R.D., Leopold K.R. (1993). Microwave spectra and structure of hydrogen cyanide-boron trifluoride: An almost weakly bound complex. J. Phys. Chem..

[17] Phillips J.A., Giesen D.J., Wells N.P., Halfen J.A., Kuntson C.C., Wrass J.P. (2005). Condensed-phase effects on the structural properties of C_6_H_5_CN−BF_3_ and (CH_3_)_3_CCN−BF_3_: IR spectra, crystallography, and computations. J. Phys. Chem. A.

[18] Phillips J.A., Halfen J.A., Wrass J.P., Knutson C.C., Cramer C.J. (2006). Large gas− solid structural differences in complexes of haloacetonitriles with boron trifluoride. Inorg. Chem..

[19] Grabowski S.J. (2014). Boron and other triel Lewis acid centers: From hypovalency to hypervalency. ChemPhysChem.

[20] Grabowski S.J. (2020). The nature of triel bonds, a case of B and Al centres bonded with electron rich sites. Molecules.

[21] Michalczyk M., Zierkiewicz W., Scheiner S. (2018). Triel-bonded complexes between TrR_3_ (Tr= B, Al, Ga; R= H, F, Cl, Br, CH_3_) and pyrazine. ChemPhysChem.

[22] Shriver D.F. (1970). Transition metal basicity. Acc. Chem. Res..

[23] Werner H. (1983). Electron-rich half-sandwich complexes—Metal bases par excellence. Angew. Chem. Int. Ed..

[24] Baker A.W., Bublitz D.E. (1966). Enthalpies of intramolecular interactions in ferrocenyl alcohols. Spectrochim. Acta.

[25] Hill E.A., Richards J.H. (1961). Carbonium ion stabilization by metallocene nuclei. III. Evidence for metal participation1. J. Am. Chem. Soc..

[26] Hill E.A., Richards J.H. (1961). Carbonium ion stabilization by metallocene nuclei. II. α-Metallocenylcarbonium ions. J. Am. Chem. Soc..

[27] Trifan D.S., Bacskai R. (1960). Metal-hydrogen bonding in metallocene compounds. J. Am. Chem. Soc..

[28] Shriver D.F. (1963). Lewis basicity of a transition metal. A boron trifluoride adduct of biscyclopentadienyltungsten dihydride. J. Am. Chem. Soc..

[29] Scott R.N., Shriver D.F., Vaska L. (1968). Lewis acid adducts of planar four-coordinated d_8_ complexes. Boron trifluoride-chlorocarbonylbis(triphenylphosphine)iridium and related systems. J. Am. Chem. Soc..

[30] Burlitch J.M., Leonowicz M.E., Petersen R.B., Hughes E.R. (1979). Coordination of metal carbonyl anions to triphenylaluminum, -gallium, and-indium and the crystal structure of tetraethylammonium triphenyl ((η^5^-cyclopentadienyl)dicarbonyliron) aluminate (Fe-Al). Inorg. Chem..

[31] Fischer R.A., Weiß J. (1999). Coordination chemistry of aluminum, gallium, and indium at transition metals. Angew. Chem. Int. Ed..

[32] Hill A.F., Owen G.R., White A.J.P., Williams D.J. (1999). The sting of the scorpion: A metallaboratrane. Angew. Chem. Int. Ed..

[33] Braunschweig H., Gruss K., Radacki K. (2007). Interaction between d-and p-block metals: Synthesis and structure of platinum–alane adducts. Angew. Chem. Int. Ed..

[34] Braunschweig H., Gruss K., Radacki K. (2008). Reactivity of Pt^0^ complexes toward gallium(III) halides: Synthesis of a platinum gallane complex and oxidative addition of gallium halides to Pt^0^. Inorg. Chem..

[35] Senda S., Ohki Y., Hirayama T., Toda D., Chen J.L., Matsumoto T., Kawaguchi H., Ttsumi K. (2006). Mono{hydrotris(mercaptoimidazolyl)borato} complexes of manganese(II), iron(II), cobalt(II), and nickel(II) halides. Inorg. Chem..

[36] Pang K., Tanski J.M., Parkin G. (2008). Reactivity of the Ni→B dative σ-bond in the nickel boratrane compounds [κ^4^-B (mim^But^)_3_] NiX (X= Cl, OAc, NCS, N_3_): Synthesis of a series of B-functionalized tris(2-mercapto-1-tert-butylimidazolyl)borato complexes, [YTm^But^]NiZ. Chem. Commun..

[37] Bikbaeva Z.M., Ivanov D.M., Novikov A.S., Ananyev I.V., Bokach N.A., Kukushkin V.Y. (2017). Electrophilic–nucleophilic dualism of nickel(II) toward Ni∙∙∙I noncovalent interactions: Semicoordination of iodine centers via electron belt and halogen bonding via σ-hole. Inorg. Chem..

[38] Ivanov D.M., Bokach N.A., Kukushkin V.Y., Frontera A. (2022). Metal centers as nucleophiles: Oxymoron of halogen bond-involving crystal engineering. Chem.–A Eur. J..

[39] Bauzá A., Frontera A. (2022). Supramolecular assemblies based on σ-hole interactions. Supramolecular Assemblies Based on Electrostatic Interactions.

[40] Katlenok E.A., Rozhkov A.V., Levin O.V., Haukka M., Kuznetsov M.L., Kukushkin V.Y. (2020). Halogen bonding involving palladium(II) as an XB acceptor. Cryst. Growth Des..

[41] Bulatov E., Eskelinen T., Ivanov A.Y., Tolstoy M., Kalenius E., Hirva P., Huukka M. (2021). Noncovalent axial I∙∙∙Pt∙∙∙I interactions in platinum(II) complexes strengthen in the excited state. ChemPhysChem.

[42] Freindorf M., Yannacone S., Oliveira V., Verma N., Kraka E. (2021). Halogen bonding involving I_2_ and d^8^ transition-metal pincer complexes. Crystals.

[43] Eliseeva A.A., Khazanova M.A., Cheranyova A.M., Aliyarova I.S., Kravchuk R.I., Oganesyan E.S., Ryabykh A.V., Maslova O.A., Ivanov D.M., Bezonsyuk S.A. (2023). Metal-involving halogen bonding confirmed using DFT calculations with periodic boundary conditions. Crystals.

[44] Li G., Stenlid J.H., Ahlquist M.S.G., Brinck T. (2020). Utilizing the surface electrostatic potential to predict the interactions of Pt and Ni nanoparticles with Lewis acids and bases—σ-lumps and σ-holes govern the catalytic activities. J. Phys. Chem. C.

[45] Zierkiewicz W., Kizior B., Michalczyk M., Jazierska A., Scheiner S. (2023). Pd and Pt Metal atoms as electron donors in σ-hole bonded complexes. Phys. Chem. Chem. Phys..

[46] Rozhkov A.V., Krykova M.A., Ivanov D.M., Novikov A.S., Sinelshchikova A.A., Volostnykh M.V., Konovalov M.A., Grigoriev M.S., Gorbunova Y.G., Kukushkin V.Y. (2019). Reverse arene sandwich structures based upon π-hole∙∙∙ [MII](d^8^ M= Pt, Pd) interactions, where positively charged metal centers play the role of a nucleophile. Angew. Chem. Int. Ed..

[47] Malenov D.P., Zarić S.D. (2018). Chelated metal ions modulate the strength and geometry of stacking interactions: Energies and potential energy surfaces for chelate–chelate stacking. Phys. Chem. Chem. Phys..

[48] Malenov D.P., Michael B.H., Zarić S.D. (2018). Influence of metal ion on chelate–aryl stacking interactions. Int. J. Quantum Chem..

[49] Goedecke C., Hillebrecht P., Uhlemann T. (2009). The Dewar–Chatt–Duncanson model reversed—Bonding analysis of group-10 complexes [(PMe_3_)_2_M–EX_3_](M= Ni, Pd, Pt; E= B, Al, Ga, In, Tl; X= H, F, Cl, Br, I). Can. J. Chem..

[50] Liu N., Li Q.Z., Scheiner S., Xie X.Y. (2022). Resonance-assisted intramolecular triel bonds. Phys. Chem. Chem. Phys..

[51] Niu Z.H., McDowell S.A.C., Li Q.Z. (2023). Triel bonds with Au atoms as electron donors. ChemPhysChem.

[52] Murcia R.A., MacLeod-Carey D., Hurtado J.J., Muñoz-Castro A. (2023). Formation of C_60_-SnI_4_ adducts. Insights of the role of σ-hole and tetrel-bonding in the strength and interaction nature from DFT calculations. Inorg. Chim. Acta.

[53] Ulloa C.O., Ponce-Vargas M., Munoz-Castro A. (2018). Formation of coinage-metal···fullerene adducts. Evaluation of the interaction nature between triangular coinage metal complexes (M_3_ = Cu, Ag, and Au) and C_60_ through relativistic density functional theory calculations. J. Phys. Chem. C.

[54] Muñoz-Castro A., Wang G., Ponduru T.T., Dias H.V.R. (2021). Synthesis and characterization of N-heterocyclic carbene-M∙∙∙OEt_2_ complexes (M = Cu, Ag, Au). Analysis of solvated auxiliary-ligand free [(NHC)M]^+^ species. Phys. Chem. Chem. Phys..

[55] Ulloa C.O., Guajardo-Maturana R., Rodríguez-Kessler P.L., Muñoz-Castro A. (2023). Nature of the dative nitrogen-coinage metal bond in molecular motors. Evaluation of NHC-M pyrazine bond (M= Cu, Ag, Au) from relativistic DFT. Inorg. Chim. Acta.

[56] Su P., Li H. (2009). Energy decomposition analysis of covalent bonds and intermolecular interactions. J. Chem. Phys..

[57] Frisch M.J., Trucks G.W., Schlegel H.B., Scuseria G.E., Robb M.A., Cheeseman J.R., Scalmani G., Barone V., Mennucci B., Petersson G.A. (2009). Gaussian 09, Revision A.02.

[58] Becke A.D. (1992). Density-functional thermochemistry. I. The effect of the exchange-only gradient correction. J. Chem. Phys..

[59] Peterson K.A., Shepler B.C., Figgen D., Stoll H. (2006). On the spectroscopic and thermochemical properties of ClO, BrO, IO, and their anions. J. Phys. Chem. A.

[60] Wilson A.K., Woon D.E., Peterson K.A., Dunning T.H. (1999). Gaussian basis sets for use in correlated molecular calculations. IX. The atoms gallium through krypton. J. Chem. Phys..

[61] Boys S.F., Bernardi F. (2002). The calculation of small molecular interactions by the differences of separate total energies. Some procedures with reduced errors. Mol. Phys..

[62] Lu T., Chen F.W. (2012). Multiwfn: A multifunctional wavefunction analyzer. J. Comput. Chem..

[63] Bader R.F.W. (1985). Atoms in molecules. Acc. Chem. Res..

[64] Lu T., Chen Q. (2021). Interaction region indicator: A simple real space function clearly revealing both chemical bonds and weak interactions. Chemistry-Methods.

[65] Mitoraj M.P., Michalak A., Ziegler T. (2009). A combined charge and energy decomposition scheme for bond analysis. J. Chem. Theory Comput..

[66] Tang Z., Song Y.L., Zhang S., Wang W., Xu Y., Wu D., Wu W., Su P.F. (2021). XEDA, a fast and multipurpose energy decomposition analysis program. J. Comput. Chem..

[67] Su P.F., Tang Z., Wu W. (2020). Generalized Kohn-Sham energy decomposition analysis and its applications. Wiley Interdiscip. Rev. Comput. Mol. Sci..

[68] Zou J.X. MOKIT Program. https://gitlab.com/jxzou/mokit.

