# Supplementary material for: Triel Bonds between BH3/C5H4BX and M(MDA)2 (X = H, CN, F, CH3, NH2; M = Ni, Pd, Pt, MDA = Enolated Malondialdehyde) and Group 10 Transition Metal Electron Donors
Source: Molecules. 2024 Apr 3;29(7):1602. doi: 10.3390/molecules29071602 (PMC11013632; doi:10.3390/molecules29071602)
Supplement: Supplementary file 1 [file molecules-29-01602-s001.zip › molecules-2923194-supplementary.pdf]

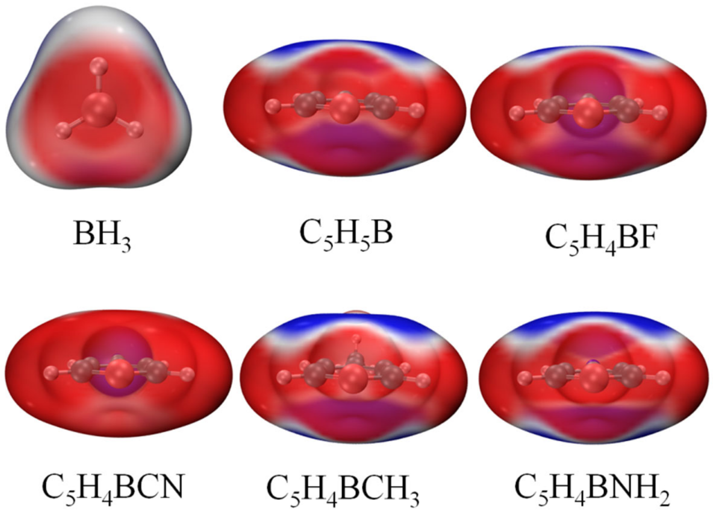

Figure S1. The MEP maps of the Lewis acids.

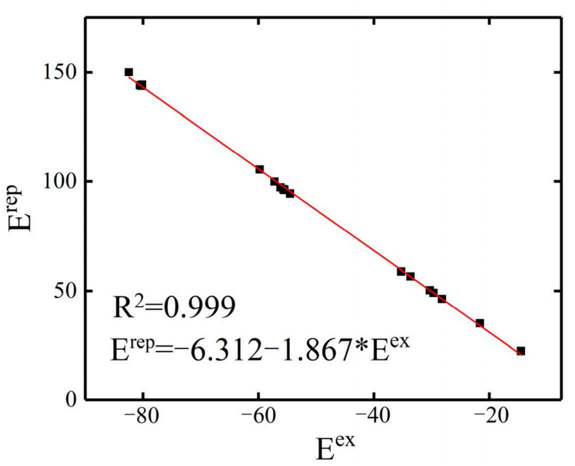

Figure S2. Linear relationship between the exchange energy (E^{ex}, kcal/mol) and the repulsion energy (E^{rep}, kcal/mol) in $BH_3/C_5H_4BX\cdots M(MDA)_2$.

Cartesian coordinates

$BH_3\cdots Ni(MDA)_2$

C	-2.52318600	-1.26460000	0.06806400
H	-3.10875000	-2.16739700	0.25804300
C	-3.20175800	-0.06274100	-0.10693500
H	-4.27831700	-0.05062400	-0.04537300
C	-2.52233400	1.11843900	-0.38002400
H	-3.10514800	2.02974900	-0.53235600
O	-1.27121900	-1.44227000	0.02674900
O	-1.26856800	1.26779500	-0.48131300
C	2.52375600	-1.26429000	0.06842800

H	3.10956400	-2.16705000	0.25791000
C	3.20213500	-0.06220500	-0.10598700
H	4.27867400	-0.04993900	-0.04410100
C	2.52258000	1.11883600	-0.37929000
H	3.10523900	2.03029700	-0.53132000
O	1.27186100	-1.44220100	0.02708400
O	1.26883200	1.26786900	-0.48134400
Ni	0.00010500	-0.08697700	-0.22563300
B	-0.00231800	1.01177900	2.39567300
H	1.02638300	0.42754100	2.51164100
H	-0.00316500	2.18764800	2.23861600
H	-1.03022900	0.42606200	2.51135900

$C_5H_4BCN\cdots Ni(MDA)_2$

C	1.60220400	-2.50649100	1.20952200
H	1.61843200	-3.08213000	2.13684100
C	1.60808300	-3.19050100	0.00003200
H	1.62883800	-4.26839500	0.00003600
C	1.60223200	-2.50649800	-1.20946200
H	1.61848000	-3.08214300	-2.13677700
O	1.57940400	-1.25043700	1.36815600
O	1.57943800	-1.25044500	-1.36810400
C	1.60219900	2.50649800	1.20950700
H	1.61842000	3.08214200	2.13682300
C	1.60808600	3.19050100	0.00001400
H	1.62884100	4.26839600	0.00001100
C	1.60224300	2.50649100	-1.20947600
H	1.61849900	3.08213000	-2.13679500
O	1.57939800	1.25044500	1.36814700
O	1.57944900	1.25043700	-1.36810900
Ni	1.53493300	-0.00000100	0.00002200
C	-1.50113500	-1.33040000	0.00001400
C	-2.89526800	-1.23227600	-0.00002100
C	-3.57526700	0.00000100	-0.00005600
C	-2.89526500	1.23227700	-0.00005600
C	-1.50113300	1.33039800	-0.00002200
H	-1.03934700	-2.30277900	0.00003900
H	-3.48943000	-2.13811200	-0.00002300
B	-0.93064700	-0.00000200	0.00000900
H	-3.48942600	2.13811400	-0.00008300
H	-1.03934200	2.30277500	-0.00002400
C	-5.00026600	0.00000300	-0.00009100
N	-6.15438100	0.00000400	-0.00011900

$C_5H_4BF\cdots Ni(MDA)_2$

C	1.41332100	-2.50714300	1.20968800
H	1.42230300	-3.08412100	2.13654500
C	1.40837100	-3.19120900	0.00001800
H	1.41231400	-4.26936000	0.00002000
C	1.41335800	-2.50714600	-1.20965300
H	1.42233600	-3.08412400	-2.13651000
O	1.40949200	-1.25152400	1.36863700
O	1.40952600	-1.25152700	-1.36860200
C	1.41332400	2.50714600	1.20968000
H	1.42228500	3.08412700	2.13653600
C	1.40837500	3.19120800	0.00000900
H	1.41232100	4.26936000	0.00000800
C	1.41336100	2.50714200	-1.20966000
H	1.42236300	3.08411700	-2.13651800
O	1.40948600	1.25152800	1.36863300
O	1.40953300	1.25152200	-1.36860500
Ni	1.37130500	-0.00000100	0.00001600
C	-1.72300800	-1.32901100	0.00006200
C	-3.12699500	-1.23060800	-0.00000100
C	-3.77015800	0.00000200	-0.00006700
C	-3.12699300	1.23061000	-0.00007500
C	-1.72300600	1.32901100	-0.00001200
H	-1.26262600	-2.30200500	0.00010900
H	-3.73831700	-2.12477800	-0.00000100
B	-1.15694700	-0.00000100	0.00004600
H	-3.73831400	2.12478200	-0.00012900
H	-1.26262200	2.30200400	-0.00001900
F	-5.13642700	0.00000300	-0.00012800

$C_5H_5B\cdots Ni(MDA)_2$

C	1.19937800	-2.51775300	1.23194400
H	1.23173900	-3.10388900	2.15341100
C	1.13310300	-3.19873000	0.01851200
H	1.11902300	-4.27706200	0.01982100
C	1.10273300	-2.52032700	-1.19295600
H	1.06910800	-3.10402300	-2.11551800
O	1.23281600	-1.26516300	1.39769000
O	1.10623400	-1.26379800	-1.36088000
C	1.15798300	2.54109400	1.22241300
H	1.18314200	3.13124900	2.14154600
C	1.07709100	3.21625800	0.00656200
H	1.04510500	4.29420800	0.00382200
C	1.05251700	2.53280800	-1.20217100

H	1.00439100	3.11221500	-2.12676300
O	1.21203900	1.28989600	1.39281300
O	1.07659700	1.27580800	-1.36532500
Ni	1.14272900	0.00896700	0.01941500
C	-2.10210400	-1.35230700	-0.29635400
C	-3.44710500	-1.25461700	0.11595300
C	-4.10105100	-0.03697600	0.32337300
C	-3.46857600	1.19573000	0.13965200
C	-2.12557200	1.32483700	-0.27048800
H	-1.64427700	-2.31594600	-0.43506200
H	-4.00037200	-2.17318800	0.27858700
B	-1.60770500	-0.00791900	-0.42459800
H	-5.13531300	-0.04907400	0.63674000
H	-4.03777800	2.10115100	0.32005000
H	-1.68453000	2.29882600	-0.39028200

$C_5H_4BCH_3$···Ni(MDA)$_2$

C	1.41354900	-2.50873300	1.23803400
H	1.39994300	-3.08963200	2.16283900
C	1.42183600	-3.19157500	0.02595900
H	1.41470200	-4.26979300	0.02506800
C	1.45455700	-2.50756800	-1.18217800
H	1.47560200	-3.08475200	-2.10886500
O	1.42390500	-1.25508400	1.39929900
O	1.46245700	-1.25123600	-1.34124600
C	1.41353300	2.50874100	1.23803000
H	1.39992500	3.08964100	2.16283400
C	1.42181400	3.19158100	0.02595400
H	1.41467500	4.26979900	0.02506100
C	1.45453100	2.50757200	-1.18218200
H	1.47556700	3.08475500	-2.10887000
O	1.42389300	1.25509200	1.39929700
O	1.46243600	1.25124000	-1.34124900
Ni	1.41108900	0.00000300	0.02909700
C	-1.72469100	-1.33019500	-0.21420000
C	-3.11826100	-1.21675900	-0.04188400
C	-3.80746300	-0.00001100	0.04005400
C	-3.11827500	1.21674500	-0.04187300
C	-1.72470600	1.33019900	-0.21418700
H	-1.26948100	-2.30433600	-0.26162300
H	-3.69524700	-2.13356700	0.03192600
B	-1.17532500	0.00000500	-0.27040400
H	-3.69527200	2.13354600	0.03194600
H	-1.26950700	2.30434500	-0.26160200

C	-5.31014300	-0.00002100	0.18338800
H	-5.80900100	-0.00002400	-0.78982900
H	-5.65749600	-0.88014600	0.72506000
H	-5.65750600	0.88010000	0.72506000

$C_5H_4BNH_2\cdots Ni(MDA)_2$

C	1.60220400	-2.50649100	1.20952200
H	1.61843200	-3.08213000	2.13684100
C	1.60808300	-3.19050100	0.00003200
H	1.62883800	-4.26839500	0.00003600
C	1.60223200	-2.50649800	-1.20946200
H	1.61848000	-3.08214300	-2.13677700
O	1.57940400	-1.25043700	1.36815600
O	1.57943800	-1.25044500	-1.36810400
C	1.60219900	2.50649800	1.20950700
H	1.61842000	3.08214200	2.13682300
C	1.60808600	3.19050100	0.00001400
H	1.62884100	4.26839600	0.00001100
C	1.60224300	2.50649100	-1.20947600
H	1.61849900	3.08213000	-2.13679500
O	1.57939800	1.25044500	1.36814700
O	1.57944900	1.25043700	-1.36810900
Ni	1.53493300	-0.00000100	0.00002200
C	-1.50113500	-1.33040000	0.00001400
C	-2.89526800	-1.23227600	-0.00002100
C	-3.57526700	0.00000100	-0.00005600
C	-2.89526500	1.23227700	-0.00005600
C	-1.50113300	1.33039800	-0.00002200
H	-1.03934700	-2.30277900	0.00003900
H	-3.48943000	-2.13811200	-0.00002300
B	-0.93064700	-0.00000200	0.00000900
H	-3.48942600	2.13811400	-0.00008300
H	-1.03934200	2.30277500	-0.00002400
C	-5.00026600	0.00000300	-0.00009100
N	-6.15438100	0.00000400	-0.00011900

$BH_3\cdots Pd(MDA)_2$

C	-2.59114900	-1.23212500	-0.13150600
H	-3.21562300	-2.12814800	-0.11462700
C	-3.23730000	0.00014500	-0.08830200
H	-4.31476400	0.00013300	-0.03198800
C	-2.59113000	1.23243500	-0.13065100
H	-3.21559300	2.12845400	-0.11315800
O	-1.34949600	-1.46633200	-0.19612400

O	-1.34947100	1.46666300	-0.19508300
C	2.59797500	-1.23207100	-0.11964400
H	3.22253200	-2.12804300	-0.10079900
C	3.24305300	0.00012000	-0.05761600
H	4.31937300	0.00009700	0.01740600
C	2.59796300	1.23235000	-0.11872700
H	3.22251400	2.12831200	-0.09919900
O	1.35770600	-1.46558800	-0.20656600
O	1.35769300	1.46591700	-0.20549400
Pd	0.00352700	0.00014700	-0.18217300
B	-0.05186800	-0.00223100	2.36169400
H	0.53282900	-1.03350200	2.45339300
H	-1.24132800	0.00143400	2.41033300
H	0.53921700	1.02527500	2.45492800

$C_5H_4BCN\cdots Pd(MDA)_2$

C	-1.42695300	2.59447100	1.23124200
H	-1.45286800	3.21325000	2.12993000
C	-1.44220300	3.24270900	-0.00007000
H	-1.47588300	4.32085300	-0.00009700
C	-1.42697000	2.59441000	-1.23134900
H	-1.45289500	3.21314300	-2.13006800
O	-1.38787100	1.34994300	1.46327700
O	-1.38789000	1.34987000	-1.46332200
C	-1.42657200	-2.59451700	1.23137400
H	-1.45241600	-3.21325600	2.13009100
C	-1.44172100	-3.24281800	0.00009300
H	-1.47525500	-4.32096700	0.00012000
C	-1.42658900	-2.59457900	-1.23122100
H	-1.45244500	-3.21336200	-2.12990700
O	-1.38769200	-1.34997200	1.46334800
O	-1.38771000	-1.35004500	-1.46325800
Pd	-1.32248600	-0.00004900	0.00001100
C	1.59507900	1.31904000	-0.00005300
C	2.98500400	1.22950500	-0.00005700
C	3.66790800	0.00013000	-0.00002900
C	2.98506900	-1.22928200	0.00000700
C	1.59515000	-1.31888700	0.00001600
H	1.14354400	2.29738800	-0.00007600
H	3.57902000	2.13576700	-0.00008400
B	0.97072900	0.00006100	-0.00001500
H	3.57913200	-2.13551300	0.00002700
H	1.14365900	-2.29725700	0.00004300
C	5.09244000	0.00016800	-0.00003600

N	6.24693600	0.00020400	-0.00004200

$C_5H_4BF \cdots Pd(MDA)_2$

C	-1.25176100	2.59454500	1.23140000
H	-1.27340900	3.21459000	2.12957600
C	-1.26133800	3.24299800	-0.00006900
H	-1.28515700	4.32146100	-0.00009600
C	-1.25178300	2.59448300	-1.23150500
H	-1.27344400	3.21448200	-2.12971300
O	-1.22381900	1.35043700	1.46364600
O	-1.22384300	1.35036200	-1.46368900
C	-1.25106200	-2.59470600	1.23153100
H	-1.27257400	-3.21471200	2.12973800
C	-1.26043800	-3.24322200	0.00009400
H	-1.28397000	-4.32169100	0.00012200
C	-1.25108200	-2.59476800	-1.23137500
H	-1.27261100	-3.21481900	-2.12955000
O	-1.22349500	-1.35057800	1.46371600
O	-1.22351900	-1.35065100	-1.46362300
Pd	-1.16060500	-0.00010100	0.00001200
C	1.78253800	1.31743000	-0.00005600
C	3.18233700	1.22784600	-0.00006300
C	3.82726200	0.00032900	-0.00003600
C	3.18253000	-1.22729000	-0.00000200
C	1.78274600	-1.31709000	0.00000900
H	1.33267900	2.29652700	-0.00007800
H	3.79315600	2.12269900	-0.00008900
B	1.16203700	0.00012200	-0.00001900
H	3.79348800	-2.12204700	0.00001700
H	1.33303400	-2.29625800	0.00003600
F	5.19601900	0.00043700	-0.00004400

$C_5H_5B \cdots Pd(MDA)_2$

C	2.58265800	1.02359000	1.23173200
H	3.20361800	1.05081100	2.12930200
C	3.23073100	1.03999200	-0.00006700
H	4.30896300	1.07404900	-0.00009500
C	2.58259400	1.02361300	-1.23183200
H	3.20350600	1.05086900	-2.12943500
O	1.33937200	0.98360200	1.46454100
O	1.33929600	0.98361500	-1.46457700
C	-2.60662700	0.96112300	1.23185600
H	-3.22803400	0.97345000	2.12944700
C	-3.25497100	0.96148300	0.00009400

H	-4.33371300	0.96920300	0.00012000
C	-2.60668700	0.96112000	-1.23169900
H	-3.22813600	0.97343300	-2.12926000
O	-1.36272300	0.95147400	1.46459500
O	-1.36279400	0.95148300	-1.46449800
Pd	-0.01100400	0.90685300	0.00001600
C	1.34664100	-2.03730600	-0.00009000
C	1.26247100	-3.43813100	-0.00007300
C	0.05021500	-4.12968500	-0.00003200
C	-1.17861000	-3.46800300	-0.00000700
C	-1.29711600	-2.06965900	-0.00001900
H	2.31887400	-1.57295900	-0.00012200
H	2.18304500	-4.01292400	-0.00009200
B	0.01738000	-1.44917600	-0.00005700
H	0.06345600	-5.21091600	-0.00002100
H	-2.08481800	-4.06518400	0.00002400
H	-2.28050000	-1.62938600	0.00000300

$C_5H_4BCH_3$···Pd(MDA)$_2$

C	1.27385900	2.59482100	-1.22983200
H	1.29493000	3.21592700	-2.12747400
C	1.27693700	3.24324500	0.00181300
H	1.29474000	4.32187300	0.00177800
C	1.26734500	2.59500800	1.23357900
H	1.28342000	3.21632100	2.13118500
O	1.25305400	1.35104500	-1.46250400
O	1.24583200	1.35130300	1.46636000
C	1.27370700	-2.59486600	-1.22983300
H	1.29475100	-3.21597400	-2.12747500
C	1.27675000	-3.24328900	0.00181300
H	1.29450000	-4.32191800	0.00177700
C	1.26719600	-2.59505300	1.23357900
H	1.28324400	-3.21636800	2.13118300
O	1.25297600	-1.35108900	-1.46250600
O	1.24575600	-1.35134700	1.46636200
Pd	1.18918900	-0.00002200	0.00183100
C	-1.77529700	1.31744400	-0.00940800
C	-3.17462600	1.21332300	-0.01376600
C	-3.87136000	0.00006500	-0.01286900
C	-3.17466300	-1.21321200	-0.01376400
C	-1.77533600	-1.31737500	-0.00940600
H	-1.32864500	2.29800900	-0.01584700
H	-3.75643700	2.13068300	-0.02044100
B	-1.16444400	0.00002500	-0.00338400

H	-3.75650100	-2.13055500	-0.02043900
H	-1.32871300	-2.29795300	-0.01584500
C	-5.38042500	0.00009300	0.02115100
H	-5.78918600	-0.88032400	-0.47571200
H	-5.76289400	0.00024900	1.04593300
H	-5.78916500	0.88037400	-0.47597100

$C_5H_4BNH_2\cdots Pd(MDA)_2$

C	1.26006700	-2.59535700	1.23201100
H	1.27723400	-3.21668500	2.12960800
C	1.26551000	-3.24361500	0.00040300
H	1.28083900	-4.32228100	0.00023400
C	1.26221300	-2.59487600	-1.23124100
H	1.28044100	-3.21575200	-2.12910700
O	1.24109500	-1.35157700	1.46500300
O	1.24449800	-1.35115000	-1.46337600
C	1.26140700	2.59479900	1.23213200
H	1.27875400	3.21606900	2.12976200
C	1.26722400	3.24311100	0.00056600
H	1.28296900	4.32177200	0.00044700
C	1.26377500	2.59443400	-1.23110600
H	1.28230300	3.21535400	-2.12893400
O	1.24191500	1.35100500	1.46506400
O	1.24541200	1.35072900	-1.46330600
Pd	1.18243700	-0.00021900	0.00091600
C	-1.78385800	-1.31271200	0.00164700
C	-3.18368400	-1.21625800	-0.00666800
C	-3.87295600	0.00077800	-0.00952300
C	-3.18351800	1.21738900	-0.00691000
C	-1.78338000	1.31384900	0.00124700
H	-1.33886900	-2.29425000	0.00573300
H	-3.76905400	-2.13130400	-0.01746800
B	-1.16310100	0.00055100	0.00143300
H	-3.76840500	2.13275700	-0.01776300
H	-1.33906100	2.29563900	0.00511400
N	-5.29269700	0.00051900	-0.08579600
H	-5.70730200	-0.82226400	0.32585500
H	-5.70772700	0.82334400	0.32534100

$BH_3\cdots Pt(MDA)_2$

C	2.59017500	1.23481300	-0.08838200
H	3.21152200	2.13062000	-0.07076200
C	3.22919000	0.00002500	-0.04838900
H	4.30673100	0.00002300	0.00840500

C	2.59018000	-1.23476100	-0.08855100
H	3.21152900	-2.13056900	-0.07105000
O	1.34514900	1.47424000	-0.15268500
O	1.34515600	-1.47418300	-0.15289700
C	-2.59304300	1.23481900	-0.08148700
H	-3.21458700	2.13049600	-0.06327500
C	-3.23077100	0.00002100	-0.02022100
H	-4.30706200	0.00001700	0.05667900
C	-2.59304900	-1.23477100	-0.08168400
H	-3.21459500	-2.13045000	-0.06361700
O	-1.34951900	1.47346100	-0.16971700
O	-1.34952500	-1.47340200	-0.16994900
Pt	-0.00201200	0.00002500	-0.14138800
B	0.03255400	-0.00048000	2.30554300
H	-0.55466200	1.02657000	2.45185400
H	-0.55230200	-1.02893100	2.45141700
H	1.22138500	0.00086400	2.41515800

$C_5H_4BCN\cdots Pt(MDA)_2$

C	1.20942100	-2.59546400	1.23379500
H	1.23431900	-3.21159800	2.13204400
C	1.22184300	-3.23630100	0.00000200
H	1.24848100	-4.31474200	-0.00000300
C	1.20950500	-2.59545100	-1.23378400
H	1.23443900	-3.21157500	-2.13203800
O	1.17731600	-1.34716200	1.47136400
O	1.17740100	-1.34714700	-1.47134100
C	1.20945200	2.59545300	1.23382100
H	1.23435400	3.21157800	2.13207500
C	1.22182700	3.23630300	0.00003500
H	1.24845900	4.31474400	0.00004100
C	1.20944800	2.59546600	-1.23375800
H	1.23437200	3.21160100	-2.13200600
O	1.17734700	1.34714900	1.47137800
O	1.17735600	1.34716400	-1.47132900
Pt	1.11087800	0.00000200	0.00001700
C	-1.78982400	-1.30919300	0.00008100
C	-3.17771200	-1.22748500	0.00005100
C	-3.86309400	-0.00000500	-0.00006000
C	-3.17771600	1.22747800	-0.00014600
C	-1.78982900	1.30919200	-0.00012800
H	-1.34595600	-2.29247600	0.00017100
H	-3.77068700	-2.13454300	0.00011300
B	-1.12173700	0.00000100	-0.00001200

H	-3.77069500	2.13453400	-0.00022900
H	-1.34596400	2.29247600	-0.00020200
C	-5.28708500	-0.00000700	-0.00008500
N	-6.44195200	-0.00001000	-0.00010600

$C_5H_4BF\cdots Pt(MDA)_2$

C	1.05425400	-2.59471600	1.23393200
H	1.07514400	-3.21201400	2.13169200
C	1.06124400	-3.23576000	-0.00001000
H	1.07816700	-4.31444300	-0.00001600
C	1.05437300	-2.59469800	-1.23394300
H	1.07531200	-3.21198300	-2.13171100
O	1.03333300	-1.34687000	1.47166600
O	1.03344100	-1.34684900	-1.47166400
C	1.05432800	2.59469500	1.23397700
H	1.07523700	3.21197500	2.13174900
C	1.06123600	3.23576300	0.00004700
H	1.07815900	4.31444500	0.00006000
C	1.05428000	2.59472300	-1.23389800
H	1.07519400	3.21202600	-2.13165400
O	1.03339000	1.34684500	1.47168900
O	1.03336600	1.34687900	-1.47164100
Pt	0.96910000	0.00000100	0.00000900
C	-1.94927900	-1.30752500	0.00012400
C	-3.34732600	-1.22589400	0.00009000
C	-3.99320300	-0.00000700	-0.00005400
C	-3.34733600	1.22588600	-0.00017100
C	-1.94929000	1.30753000	-0.00015100
H	-1.50758600	-2.29178800	0.00024300
H	-3.95815700	-2.12087000	0.00017300
B	-1.28530200	0.00000500	-0.00000300
H	-3.95817500	2.12085700	-0.00027800
H	-1.50760600	2.29179600	-0.00025200
F	-5.36410500	-0.00001200	-0.00008100

$C_5H_5B\cdots Pt(MDA)_2$

C	0.83251500	-2.59398400	1.23422600
H	0.85299700	-3.21190300	2.13166400
C	0.84084800	-3.23482300	0.00015300
H	0.85820400	-4.31353500	0.00020000
C	0.83255700	-2.59409600	-1.23397800
H	0.85318100	-3.21209700	-2.13135700
O	0.81054900	-1.34653700	1.47240200
O	0.81055900	-1.34667000	-1.47227500

C	0.83029400	2.59477800	1.23399200
H	0.85034400	3.21279300	2.13137400
C	0.83811100	3.23551500	-0.00013700
H	0.85455600	4.31424200	-0.00018100
C	0.83038300	2.59467300	-1.23421100
H	0.85039100	3.21261400	-2.13164500
O	0.80933900	1.34733500	1.47228200
O	0.80948300	1.34720900	-1.47239400
Pt	0.74657200	0.00030800	0.00000000
C	-2.17699500	-1.31206500	-0.00028400
C	-3.57430500	-1.21998300	-0.00031500
C	-4.25429600	-0.00177700	-0.00005600
C	-3.57535100	1.21701100	0.00025100
C	-2.17811900	1.31029800	0.00031800
H	-1.73283400	-2.29532800	-0.00052100
H	-4.15922700	-2.13449000	-0.00054700
B	-1.52433700	-0.00060300	0.00003400
H	-5.33597600	-0.00224000	-0.00009400
H	-4.16105700	2.13101600	0.00044500
H	-1.73481100	2.29394600	0.00059000

$C_5H_4BCH_3\cdots Pt(MDA)_2$

C	-1.07174300	-2.59438100	-1.23271100
H	-1.09257500	-3.21243900	-2.13007600
C	-1.07457700	-3.23540500	0.00132800
H	-1.08782600	-4.31416800	0.00128600
C	-1.06637000	-2.59454500	1.23544700
H	-1.08327100	-3.21276200	2.13278600
O	-1.05487900	-1.34689900	-1.47081600
O	-1.04873800	-1.34710900	1.47368300
C	-1.07171500	2.59439900	-1.23268400
H	-1.09255400	3.21246500	-2.13004300
C	-1.07455300	3.23541100	0.00136200
H	-1.08779600	4.31417400	0.00133000
C	-1.06635800	2.59453700	1.23547400
H	-1.08324200	3.21274500	2.13281900
O	-1.05486000	1.34692000	-1.47080100
O	-1.04873400	1.34709800	1.47369600
Pt	-0.98887400	0.00000200	0.00135200
C	1.93953100	-1.30638300	-0.00856700
C	3.33649400	-1.21100800	-0.01358300
C	4.03576000	-0.00001100	-0.01289200
C	3.33650600	1.21099200	-0.01364700
C	1.93954400	1.30638300	-0.00863500

H	1.50135900	-2.29240500	-0.01458400
H	3.91766100	-2.12919100	-0.02065800
B	1.27995400	0.00000300	-0.00291700
H	3.91768200	2.12917000	-0.02077100
H	1.50138200	2.29240800	-0.01470700
C	5.54466400	-0.00001800	0.02104600
H	5.92837400	0.00000800	1.04577600
H	5.95389300	-0.88059700	-0.47586000
H	5.95390200	0.88053100	-0.47590700

$C_5H_4BNH_2 \cdots Pt(MDA)_2$

C	-1.06065600	-2.59402200	-1.23372600
H	-1.07891100	-3.21188000	-2.13128700
C	-1.06205300	-3.23537500	0.00030700
H	-1.07101000	-4.31418700	0.00011900
C	-1.05981300	-2.59449700	1.23431200
H	-1.07791800	-3.21278300	2.13160000
O	-1.04937200	-1.34651500	-1.47126800
O	-1.04768800	-1.34691800	1.47271000
C	-1.05964200	2.59425400	-1.23377700
H	-1.07768100	3.21210600	-2.13134700
C	-1.06085000	3.23562700	0.00024600
H	-1.06946900	4.31444200	0.00004000
C	-1.05879600	2.59477600	1.23426600
H	-1.07666000	3.21309000	2.13153900
O	-1.04877700	1.34674200	-1.47130100
O	-1.04707800	1.34720100	1.47269600
Pt	-0.98449200	0.00011300	0.00084200
C	1.94718800	-1.30288300	-0.00044100
C	3.34498000	-1.21490400	-0.00723100
C	4.03651700	-0.00053900	-0.00945800
C	3.34534000	1.21410700	-0.00712700
C	1.94764200	1.30251200	-0.00028300
H	1.51071200	-2.28976800	0.00309300
H	3.92962800	-2.13088900	-0.01706600
B	1.28017000	-0.00009400	-0.00020200
H	3.93033000	2.12987100	-0.01696200
H	1.51140800	2.28951200	0.00332100
N	5.45771700	-0.00068600	-0.08384200
H	5.86971100	0.82162300	0.33198400
H	5.86952300	-0.82312000	0.33194800
